# Quantifying the Mitigating Effects of Whole-Breast Radiotherapy and Systemic Treatments on Regional Recurrence Incidence Among Breast Cancer Patients

**DOI:** 10.1245/s10434-020-08356-2

**Published:** 2020-03-20

**Authors:** Julia E. C. van Steenhoven, Anne Kuijer, Marissa C. van Maaren, Marleen Roos, Sjoerd G. Elias, Paul J. van Diest, Sabine Siesling, Marjolein L. Smidt, Liesbeth J. Boersma, Thijs van Dalen

**Affiliations:** 1 Department of Surgery, Diakonessenhuis Utrecht, Utrecht, The Netherlands; 2 Department of Pathology, University Medical Center Utrecht, Utrecht, The Netherlands; 3 Department of Surgery, St. Antonius Hospital, Nieuwegein, The Netherlands; 4 Department of Research, Netherlands Comprehensive Cancer Organisation, Utrecht, The Netherlands; 5 Department of Health Technology and Services Research, Technical Medical Centre, University of Twente, Enschede, The Netherlands; 6 Department of Internal Medicine, Diakonessenhuis Utrecht, Utrecht, The Netherlands; 7 Department of Epidemiology, Julius Center for Health Sciences and Primary Care, University Medical Center Utrecht, Utrecht University, Utrecht, The Netherlands; 8 Department of Surgery, University Medical Center Maastricht, Maastrischt, The Netherlands; 9 Department of Radiation Oncology (Maastro), GROW School for Oncology and Developmental Biology, Maastricht University Medical Centre+, Maastricht, The Netherlands

## Abstract

**Background:**

Despite the potential for residual lymph node metastases after a negative or positive sentinel lymph node biopsy (SLNB), breast cancer patients rarely experience regional recurrences (RRs). This study aimed to quantify the effects of nonsurgical treatments on RR incidence among SLNB-negative (SLNB N0) breast cancer patients.

**Methods:**

All primary SLNB N0-staged breast cancer patients with a diagnosis between 2005 and 2008 and 5-year follow-up data on recurrences were selected from the Netherlands Cancer Registry. The cumulative incidence function (CIF) for RR was calculated as the first event at 5 years, taking into account any other first-event (local or distant recurrence, contralateral breast cancer, or death) as competing risk. Cox regression analysis was used to model the cause-specific hazard of RR developing as the first event to quantify the effect of adjuvant systemic therapy and whole-breast radiotherapy (RT) on RR incidence at 5 years.

**Results:**

The study included 13,512 patients. Of these patients, 162 experienced an RR. The CIF of RR at 5 years was 1.3% (95% confidence interval [CI], 1.1–1.5%), whereas the CIFs for death and other events were 4.4% and 9.5%, respectively. Cox regression analysis showed hazard ratios (HRs) of 0.46 (95% CI 0.33–0.64), 0.31 (95% CI 0.18–0.55), and 0.40 (95% CI 0.24–0.67) respectively for patients treated by RT as a routine part of breast-conserving therapy (BCT), chemotherapy, and hormonal therapy.

**Conclusion:**

RT as routine part of BCT, chemotherapy, and hormonal therapy independently exerted a mitigating effect on the risk for the development of RR. The three methods at least halved the risk.

**Electronic supplementary material:**

The online version of this article (10.1245/s10434-020-08356-2) contains supplementary material, which is available to authorized users.

Sentinel lymph node biopsy (SLNB) has replaced axillary lymph node dissection (ALND) as a minimally invasive staging procedure for patients with invasive breast cancer. Whereas meta-analyses documented a false-negative rate of 5% to 7% for the SLNB procedure, as proven for patients undergoing SLNB followed by ALND, only 0.3% to 0.6% of the patients staged as N0 by SLNB will experience an axillary recurrence.^1^–^5^ Likewise, patients who have tumor-positive sentinel lymph nodes (SLN N+) but do not undergo completion ALND also will rarely experience axillary recurrence. The Z0011 study and the After Mapping of the Axilla: Radiotherapy Or Surgery (AMAROS) trial study showed that a 27% to 33% chance of additional lymph node metastases translates into only 1.5% of patients experiencing regional metastases without further axillary surgery.^6^–^8^ The discrepancy between the frequent presence of additional lymph node metastases in both SLN N0 and SLN N+ patients and the rare event of regional recurrence (RR) is intriguing and commonly attributed to the effects of additional nonsurgical treatments such as radiotherapy (RT), chemotherapy (CT) and hormonal therapy (HT).

In a previous study, we evaluated the “natural course” RR development in patients who underwent ablative surgery, had a tumor-free SLNB, and did not receive additional nonsurgical treatments.^9^ The observed risk for the development of an RR (2.4%) was less than half the false-negative rate (5–7%) of the procedure, implying that residual lymph node metastases do not automatically develop into RR when left untreated. As such, this observation closely resembled the historical findings of the National Surgical Adjuvant Breast and Bowel Project (NSABP)-04 trial, in which less than half of the patients with nodal metastases (based on the incidence of metastases in the ALND arm) experienced clinically apparent RR although none of these patients had received adjuvant systemic therapy or RT.^10^

The current study aimed to address the contribution of nonsurgical treatments that were not given primarily to reduce the risk for the development of RR. For this purpose, we quantified the effects of RT on the breast and systemic treatments in a large population-based cohort of SLN N0 breast cancer patients.

## Methods

### Study Design and Patients

A nationwide cohort study was conducted using data of the Netherlands Cancer Registry (NCR). The NCR is a national, population-based cancer registry containing information on patient, tumor, and treatment characteristics. Event data (e.g., local recurrences (LR), RR, and distant metastases (DM)) within the first 5 years after primary breast cancer treatment had been collected directly from the patients’ files by NCR registrars.^11^ Data on vital status and date of death or last observation were derived through linkage with the Municipal Personal Records database. The Committee of Privacy of the NCR approved the use of the data for this study.

All patients who had primary unilateral invasive breast cancer without DM diagnosed between 1 January 2005 and 31 December 2008 who underwent surgery, including SLNB, at the time of diagnosis were selected from the NCR. Patients not undergoing SLNB and patients with a positive SLNB were excluded from the study. We used this cohort of SLNB N0 patients to quantify the hypothesized mitigating effects of RT and systemic treatments on the regional recurrence risk. By using this subset of node-negative patients, confounding by additional axillary RT or axillary surgery could be excluded.

To address the impact of routine RT on the breast, patients who received RT after mastectomy and patients who did not receive RT after breast-conserving therapy (BCT) were excluded from the analysis because these treatment strategies were not routine practice during the study period. Hence, all the patients in the study cohort undergoing BCT were treated by RT of the breast, and all patients undergoing mastectomy did not receive RT. Other exclusion criteria were macro- or microscopic tumor residue after the final surgery of the primary tumor, patients who received neoadjuvant systemic therapy, and patients who underwent ALND.

The following patient and tumor characteristics were collected: age, histologic type (ductal, lobular, mixed ductal/lobular, or other), pathologic tumor size (pT), histologic grade (Bloom Richardson 1/2/3), multifocality (yes/no), hormone receptor (HR) status (estrogen receptor (ER)/progesterone receptor (PR)), human epidermal growth factor receptor 2 (HER2) status, intrinsic subtype (HR+/HER2–, HR+/HER2+, HR–/HER2+, HR–/HER2–), operative treatment (mastectomy/BCT), RT (yes/no), CT (yes/no), HT (yes/no), and trastuzumab (yes/no).

During the study period, the national breast cancer clinical guideline of 2005 was effective. According to this guideline, systemic therapy (CT and/or HT for ER + patients) was advised for N0 patients with unfavorable clinicopathologic features (tumors > 3 cm, grade 3 tumors (unless < 1 cm), or grade 2 tumors > 2 cm) as well as for young patients (age < 35 years). The HER2 + patients were advised to receive trastuzumab in addition to adjuvant CT. Standard assessment of HER2 status was implemented in the Netherlands in mid-2005, whereas treatment with trastuzumab was reimbursed from 2006 onward.

### Definitions of End Points

The primary end point of the current study was RR as the first event, defined as recurrence of breast cancer in ipsilateral regional lymph nodes (e.g., axillary, infra-/supraclavicular or in the internal mammary chain).^12^ Local recurrence (LR) was defined as the occurrence of breast cancer or ductal carcinoma in situ in the ipsilateral breast or in the skin or subcutaneous tissue of the ipsilateral chest wall, and CLC was defined as the occurrence of invasive breast cancer in the contralateral breast.

Follow-up assessment began at the date of diagnoses plus 91 days and ended with any type of recurrence (event), death (censored), or date of the last follow-up visit (censored). The first event and any additional events occurring within 91 days after the first event were included for analyses (e.g., for patients who presented with DM after which RR was diagnosed during further examination within 91 days, both events were included in the analyses).

### Statistical Analysis

Distribution of baseline characteristics is presented in percentages. The cumulative incidence function (CIF) of RR as the first event at 5 years was calculated with death and any other type of event (LR, CLC, DM) as competing events. As a reference, the CIFs of death and the CIF of any other type of event (LR, CLC, DM) were calculated as well. Cumulative 5-year RR rates in relation to clinicopathologic and treatment characteristics were assessed through Kaplan–Meier estimates, and accompanying 95% confidence intervals (CIs) were calculated. Univariable analyses was performed using the log-rank test.

Multivariable Cox proportional hazards regression analysis was used to model the cause-specific hazard of RR developing as the first event within 5 years in order to quantify the effects of nonsurgical treatments on RR risk. From a clinical perspective, we included all clinicopathologic characteristics (i.e., age, grade, hormone receptor status, multifocality, histologic subtype) and nonsurgical treatments (RT, CT, HT, trastuzumab) in the analysis because all variables potentially influence RR risk. To deal with competing events, patients were censored at the date of death or other event (LR, CLC, DM). Multivariable analyses were repeated with calculation of the cause-specific hazards for death, LR, CLC, and DM as well.

Besides the variable regarding HER2 status and trastuzumab, we performed complete case analyses. Statistical analysis was performed using STATA version 14.2 (StataCorp, TX, USA). A *p* value lower than 0.05 was considered statistically significant.

## Results

### Baseline Characteristics

From the NCR, 34,734 patients with a diagnosis of primary unilateral invasive breast cancer treated surgically between 2005 and 2008 and complete 5-year follow-up assessment of recurrences were identified. The exclusion criteria ruled out patients who did not undergo SLNB (*n* = 12,318), those with a positive SLNB (*n* = 6791), those who had a mastectomy and received local RT (*n* = 228), those who had BCT but did not receive RT of the breast (*n* = 235), patients who underwent ALND (*n* = 1091), patients with a pT4 tumor (*n* = 94), and patients with macro- or microscopic tumor residue after final surgery (*n* = 465) (Fig. S1). This resulted in a study population of 13,512 patients.

The mean age of the study population was 59 ± 12 years (Table 1). The pathologic tumor size was classified as T1a–c in 76% of the patients. Of all the patients, 72% (*n* = 9674) underwent BCT and received routine RT of the breast, whereas 28% of the patients (*n* = 3838) underwent mastectomy. Adjuvant CT was administered to 22% (*n* = 3010), adjuvant HT to 25% (*n* = 3424), and CT in addition to HT to 12% (*n* = 1617) of the patients. In the patient categories with a guideline-directed indication for systemic treatment in the absence of lymph node metastases (i.e., patients younger than 70 years with large tumors [> 3 cm], grade 3 tumors, or grade 2 tumors [> 2 cm]), respectively 71%, 70% and 61% of the patients received adjuvant CT and 82%, 80%, and 86% received HT. Furthermore, among the patients younger than 70 years with information regarding HER2 status, 46% of those classified as HER2 + received trastuzumab.

**Table 1:** Patient, tumor, and treatment characteristics of the 13,512 primary breast cancer patients who had surgery between 2005 and 2008 and were staged as N0 according to sentinel lymph node biopsy

	*n*	%^a^
Mean age (years)	59 ± 12	–
Age (years)		
< 35	209	2
35–49	2724	20
50–59	3856	29
60–69	3706	27
≥ 70	3017	22
Histologic type		
Ductal	11,016	81
Lobular	1221	9
Mixed	496	4
Other^b^	779	6
Tumor size (T stage)		
T1a/1M	790	6
T1b	2847	21
T1c	6565	49
T2	3273	24
T3	37	0
Grade		
1	3785	28
2	5606	42
3	3566	26
Unknown	555	4
Multifocality		
No	12,005	89
Yes	1335	10
Unknown	172	1
Intrinsic subtype		
HR+/HER2–	9025	67
HR+/HER2+	957	7
HR–/HER2+	549	4
HR–/HER2–	1461	11
Unknown	1520	11
RT of the breast^c^		
No	3838	28
Yes	9674	72
Chemotherapy		
No	10,502	78
Yes	3010	22
Hormonal therapy		
No	10,088	75
Yes	3424	25
HER2 & trastuzumab		
HER2– & no trastuzumab	10,956	81
HER2+ & no trastuzumab	923	7
HER2+ & trastuzumab	598	4
Unknown^d^	1035	8

HR, hormone receptor; HER2, human epidermal growth factor receptor 2

^a^Percentages may not add up to 100% due to rounding

^b^Histologic tumor subtype “other” (e.g., mucinous, medullary, metaplastic carcinoma)

^c^All patients who received radiotherapy (RT) of the breast were treated with breast-conserving therapy, and patients not receiving RT of the breast were treated with mastectomy

^d^The majority of patients in the “unknown” category were diagnosed in earlier years since standard HER2 testing and treatment with trastuzumab were only routinely implemented starting September 2005

### Regional Recurrence Incidence as the First Event Within 5-Years

The number of patients who experienced an RR was 162. The CIF of RR as the first event within 5 years was 1.3% (95% CI 1.1–1.5). The CIF for death within 5 years was 4.4% (95% CI 4.0–4.8), and for LR, CLC, or DM as the first event, the CIF was 9.5% (95% CI 9.0–10.0).

Of the 162 patients, 82 (50%) experienced an isolated RR as the first event.

For 24 patients (15%), RR was diagnosed simultaneously with LR, whereas for 42 patients (26%), RR occurred simultaneously with DM, and for 14 patients (9%), RR was diagnosed simultaneously with LR and DM (Table 2).

**Table 2:** Site of the first event for the 13.512 breast cancer patients who underwent surgery between 2005 and 2008 and who were staged as N0 according to sentinel lymph node biopsy

Site of first event	Total	% of all events
No. of events	1338	
Isolated events		
RR	82	6
LR	203	15
CLC	414	31
DM	516	39
Two simultaneous events		
RR, LR	24	2
RR, DM	42	3
LR, CLC	9	1
LR, DM	30	2
CLC, DM	2	0.1
Three simultaneous events		
RR, LR, DM	14	1
LR, CLC, DM	2	0.1

RR, regional recurrence; LR, local recurrence; CLC, contralateral breast cancer; DM, distant metastasis

### Treatment Effects—Univariable Analysis

The cumulative incidence of an RR as the first event for the patients who underwent BCT and received RT as part of their routine treatment was 1.0%, whereas it was 2.3% for the patients treated with mastectomy (*p *<0.001). The cumulative incidence of RR as the first event for all the patients who had received CT was 1.3% versus 1.4% for those not treated with CT (*p* = 0.93). The cumulative incidence of RR as the first event for the patients who received HT versus the patients who did not was 1.1% versus 1.5%, respectively (*p* = 0.20). For the HER2 + patients who received trastuzumab, the cumulative RR as the first event was 1.2% compared with 2.3% for the HER2 + patients who did not receive trastuzumab (*p* = 0.10*)* (Table 3).

**Table 3:** The regional recurrence incidence as the first event within 5 years according to clinicopathologic and treatment factors of the 13,512 breast cancer patients who had surgery between 2005 and 2008 and were staged as N0 according to sentinel lymph node biopsy

	*n*	Absolute no. of RRs	RR%^a^	95% CI
Total	13,512	162	1.4	
Age (years)				
< 35	209	4	2.1	0.8–5.5
35–49	2724	49	2.0	1.5–2.6
50–59	3856	48	1.5	1.8–2.0
60–69	3706	33	1.0	0.7–1.4
> 70	3017	28	1.1	0.7–1.5
Histologic type				
Ductal	11,016	148	1.5	1.3–1.8
Lobular	1221	9	0.9	0.5–1.8
Mixed	496	4	0.9	0.33–2.3
Other^b^	779	1	0.15	0.02–1.1
Tumor size (T stage)				
T1a/1M	790	5	0.7	0.3–1.7
T1b	2847	19	0.90	0.5–1.4
T1c	6565	86	1.5	1.2–1.9
T2	3273	52	1.7	1.3–2.3
T3	37	–	–	–
Grade				
1	3785	19	0.6	0.4–1.0
2	5606	77	1.6	1.3–2.0
3	3566	61	1.9	1.5–2.4
Unknown	550	5	–	–
Multifocality				
No	12,005	134	1.3	1.1–1.5
Yes	1335	26	2.1	1.5–3.1
Unknown	172	2	1.2	0.3–4.8
Hormone receptor status				
Negative	2295	42	2.0	1.5–2.7
Positive	11,140	119	1.2	0.9–1.4
Unknown	77	1	1.4	1.2–2.2
RT of the breast				
No	3838	76	2.3	1.8–2.9
Yes	9674	86	1.0	0.8–1.3
Chemotherapy				
No	10,502	125	1.4	1.2–1.7
Yes	3010	37	1.3	1.0–1.8
Hormonal therapy				
No	10,088	128	1.5	1.2–1.8
Yes	3424	34	1.1	0.8–1.5
HER2 & trastuzumab				
HER2– & no trastuzumab	10,956	129	1.4	1.1–1.6
HER2+ & no trastuzumab	923	18	2.3	1.4–3.6
HER2+ & trastuzumab	598	7	1.2	0.6–2.6
Unknown^c^	1035	8	0.9	0.5–1.8

RR, regional recurrence; CI, confidence interval; RT, ratiotherapy; HER2, human epidermal growth factor receptor 2

^a^Represents Kaplan–Meier estimates

^b^Histologic tumor subtype “other” (e.g., mucinous, medullary, metaplastic carcinoma)

^c^Category “unknown” consists mostly of unknown treatment methods, missing in earlier years due to standard HER2 testing and treatment with trastuzumab but routinely implemented after September 2005

### Treatment Effects—Multivariable Analysis

**Fig. 1:**
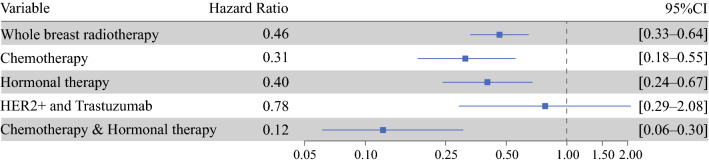
The quantitative effects of nonsurgical treatments on regional recurrence (RR) incidence as the first event within 5 years after the 13,512 breast cancer patients had surgery between 2005 and 2008 and were staged as N0 according to sentinel lymph node biopsy. Hazard ratios were assessed using multivariable Cox proportional hazards regression analyses adjusted for all clinicopathologic characteristics (age, grade, tumor size, histologic subtype, multifocality, hormone receptor status) and treatment characteristics (e.g., radiotherapy of the breast, endocrine therapy, adjuvant chemotherapy and human epidermal growth factor receptor-2 [HER2] receptor status, and trastuzumab)

Multivariable Cox regression analyses showed a significant impact of nonsurgical treatment methods on RR as the first event within 5 years. The patients treated with RT as part of BCT had a lower risk for development of RR as the first event than the patients who underwent mastectomy (hazard ratio [HR], 0.46; 95% CI 0.33–0.64). The administration of adjuvant CT and HT was significantly associated with a lower risk for development of RR as the first event (HR 0.31; 95% CI 0.18–0.55 and HR 0.40; 95% CI 0.24–0.67, respectively) (Fig. 1). The effect of combination CT/HT and all treatments combined (RT, CT, and HT) resulted in HRs of 0.12 (95% CI 0.06–0.30 and 0.05 (95% CI 0.015–0.15), respectively (Table 4). Treatment with trastuzumab had no significant impact on RR as the first event (HR 0.78; 95% CI 0.29–2.08) (Table 4).

**Table 4:** Quantitative effects of nonsurgical treatments and clinicopathologic factors on regional recurrence (RR) incidence as the first event within 5-years after the 13,512 breast cancer patients had surgery between 2005 and 2008 and were staged as N0 according to sentinel lymph node biopsy^a^

		Multivariable	
	*n*	HR^b^	95% CI
RT of the breast			
No	3838	Ref	–
Yes	9674	0.46	0.33–0.64
Chemotherapy			
No	10,502	Ref	–
Yes	3010	0.31	0.18–0.55
Hormonal therapy			
No	10,088	Ref	–
Yes	3424	0.40	0.24–0.67
HER2 & trastuzumab			
HER2– & no trastuzumab	10,956	1.0	0.58–1.76
HER2+ & no trastuzumab	923	Ref	–
HER2+ & trastuzumab	598	0.78	0.29–2.08
Unknown^c^	1035	0.71	0.30–1.68
Age (years)			
< 35	209	2.14	0.73–6.21
35–49	2724	1.49	0.99–2.47
50–59	3856	Ref	–
60–69	3706	0.62	0.39–0.99
> 70	3017	0.50	0.30–0.84
Histologic type			
Ductal	11,016	Ref	–
Lobular	1221	0.52	0.25–1.08
Mixed	496	0.59	0.22–1.62
Other^d^	779	0.12	0.02–0.88
Tumor size (T stage)			
T1a/1M	745	Ref	–
T1b	2851	1.26	0.42–3.75
T1c	6575	2.84	1.03–7.86
T2	3273	4.74	1.64–13.68
Grade			
1	3785	Ref	–
2	5606	2.96	1.78–4.95
3	3566	4.96	2.62–9.39
Multifocality			
No	12,005	Ref	–
Yes	1335	1.51	0.96–2.36
Hormone receptor status			
Negative	2295	0.99	0.57–1.67
Positive	11,140	Ref	–

Subjects (*n* = 12,702), number of missings (*n* = 810), events (*n* = 154)

^a^Hazard ratios were assessed using multivariable Cox proportional hazards regression analyses adjusted for all clinicopathologic and treatment characteristics (e.g., radiotherapy of the breast, endocrine therapy, adjuvant chemotherapy and human epidermal growth factor receptor 2 [HER2] receptor status, and trastuzumab)

^b^Category “unknown” consists mostly of unknown treatment methods, missing in earlier years due to standard HER2 testing and treatment with trastuzumab but routinely implemented after September 2005

^c^Histologic tumor subtype “other” (e.g., mucinous, medullary, metaplastic carcinoma)

### Multivariable Analysis—Competing Events

Table 5 lists the HRs of the nonsurgical treatments according to the 5-year probability of LR, CLC, DM, or death as a first event, adjusted for all clinicopathologic characteristics. Treatment with whole-breast RT was associated with a lower LR risk as a first event compared with patients who undergo mastectomy, but it was shown to have no significant impact on the risk for development of DM or CLC as the first event. Treatment with adjuvant CT was associated with a lower risk for LR as the first event and a lower risk for the development of DM compared with no CT treatment. Treatment with HT was associated with a lower risk of LR, CLC, or DM as the first event than no HT treatment, with the strongest association seen with the risk for development of CLC as the first event.

**Table 5:** Quantitative effects of nonsurgical treatments on LR, CLC, DM, and death within 5-years after the 13,512 breast cancer patients had surgery between 2005 and 2008 and were staged as N0 according to sentinel lymph node biopsy

	Multivariable				
		LR	CLC	DM	Death
	*n*	HR^a^ (95% CI)	HR^a^ (95% CI)	HR^a^ (95% CI)	HR^a^ (95%CI)
RT of the breast					
No	3838	Ref	Ref	Ref	Ref
Yes	9674	0.73 (0.56–0.95)	0.82 (0.65–1.03)	0.91 (0.75–1.09)	0.62 (0.52–0.74)
CT					
No	10,502	Ref	Ref	Ref	Ref
Yes	3010	0.46 (0.28–0.74)	0.70 (0.44–1.13)	0.56 (0.43–0.74)	0.62 (0.42–0.94)
HT					
No	10,088	Ref	Ref	Ref	Ref
Yes	3424	0.44 (0.29–0.67)	0.38 (0.26–0.56)	0.74 (0.58–0.95)	0.79 (0.61–1.02)
HER2 & TT					
HER2– & no TT	10,956	0.94 (0.61–1.45)	1.13 (0.73–1.74)	0.64 (0.49–0.84)	1.33 (0.93–1.88)
HER2+ & no TT	923	Ref	Ref	Ref	Ref
HER2+ & TT	598	0.94 (0.42–2.10)	0.64 (0.25–1.68)	0.58 (0.37–0.90)	0.84 (0.35–1.99)
Unknown^b^	1035	0.56 (0.28–1.10)	1.18 (0.69–2.01)	0.70 (0.47–1.04)	1.19 (0.75–1.86)

LR, local recurrence; CLC, contralateral breast cancer; DM, distant metastasis; HR, hazard ratio; CI, confidence interval; RT, radiotherapy; CT, chemotherapy; HT, hormonal therapy; HER2, human epidermal growth factor receptor 2; TT, trastuzumab

^a^Hazard ratios assessed using multivariable Cox proportional hazards regression analyses adjusted for all clinicopathologic characteristics (e.g., grade, size, age, histologic subtype, multifocality, hormone receptor status) and treatment characteristics

^b^Category “unknown” consists mostly of unknown treat methods, missing in earlier years due to standard HER2 testing and treatment with trastuzumab but routinely implemented after September 2005

### Clinicopathologic Factors Associated with Regional Recurrence Risk

Besides the effects of various treatment methods, larger primary tumor size and higher grade were strongly associated with an increased 5-year RR risk (Table 4), whereas histology other than ductal or lobular carcinoma was associated with lower RR risk. Age was inversely related to the risk for the development of an RR, Patients older than 70 years had a decreased risk for the development of an RR (HR 0.50; 95% CI 0.30–0.84).

## Discussion

In this population-based study of early breast cancer patients staged N0, the use of whole breast RT as routine part of BCT, HT or CT was associated with a lower risk of RR developing as the first event within 5 years after diagnosis. Besides the nonsurgical treatment methods, younger age, larger tumor size, and higher grade were associated with higher RR incidence. To our knowledge, this is the first study to report the magnitude of these effects in a large population-based cohort.

The patients who received RT as part of BCT had a significantly lower risk for the development of RR (HR 0.46; 95% CI 0.33–0.64). The mitigating effect of local RT on the risk for the development of RR has been described previously.^5^,^13^–^15^ A prospective study by Van Wely et al.^14^ showed a disproportionately high number of axillary recurrences after negative SLNB for patients who underwent ablative surgery and attributed this observation to the absence of external beam radiation therapy (EBRT) for patients who undergo a mastectomy. The hypothesis that EBRT reduces RR risk was supported by a meta-analysis performed by the same author^5^ and is line with other studies.^13^–^15^

The presence of an effect of RT to the breast on the risk for the development of RR may be explained by the incidental irradiation of the lower axilla by local RT. Studies have demonstrated that the SLN site is radiated in 79% to 94% of patients undergoing conventional two-dimensional (2D) irradiation of the breast.^16^,^17^ Radiation techniques have evolved, and 3D CT planning usually was applied in the Netherlands during the period that the patients in this study were treated. Even with 3D CT-planning techniques, the 95% isodose line still has been found to encompass 55% of the axillary levels 1 and 2 lymph node anatomic volume,^18^ and it has been hypothesized that for 76% of patients, the site of the SLN received an elective radiation dose.^19^ However, in the current era, radiation techniques have improved further, with such procedures as intensity-modulated radiotherapy (IMRT), volumetric modulated-arc therapy (VMAT),^20^ and protontherapy, resulting in even more conformal dose distributions around strictly defined target volumes.^21^ These techniques reduce any accidental dose to the axillary nodes considerably and are expected to reduce side effects due to lower doses to healthy tissues. Because we have shown a significant effect of accidental axillary dose, axillary recurrences may increase in the current era due to these new radiation techniques.

The current study, was able to address the effects of adjuvant systemic therapies on the RR risk for a substantial proportion of patients. The beneficial effects of adjuvant CT and HT were strong (HR 0.31; 95% CI 0.18–0.55 and HR 0.40; 95% CI 0.24–0.67, respectively), and the combined effect was even stronger (HR 0.12; 95% CI 0.06–0.30).

During the last decades, systemic treatment methods have evolved extensively, leading to improved survival^22^,^23^ and better locoregional control.^22^–^26^ The overview of the Early Breast Cancer Trialists’ Collaborative Group **(**EBCTCG) illustrated that treatment with tamoxifen diminished the LR rates by nearly 50% compared with placebo,^23^ and the use of aromatase inhibitors decreased the LR rates even further.^27^ Treatment with cytotoxic CT and targeted therapy improved locoregional control to an even greater extent.^28^–^31^

An effect of trastuzumab on the recurrence risk could not be demonstrated in this study. The absence of this effect may be explained by the fact that HER2-receptor status testing was not routinely applied, and only a small proportion of HER2+ patients received this type of treatment at that time. Besides the effect of local RT and systemic therapies, our results demonstrate that tumor malignancy grade, tumor size, histologic subtype, and age are associated with RR risk.

Although nonsurgical therapies contribute to a lower RR risk, the observed 1.4% RR rate for the study population still was much lower than the previously reported false-negative rate of the SLNB procedure (i.e., 5–7% in the literature).

In a previous study, we concluded that not all residual lymph node metastases will develop into clinically overt RR when left untreated.^9^ As such, this finding coincides with randomized trials in the past comparing ALND with no-ALND and reporting much lower RR rates based on the incidence of nodal metastases in the ALND arm of the study.^10^ On the one hand, this may be explained by the natural course of nodal metastases, but on the other hand, it also may be due to the fact that DMs occur before the nodal metastases become clinically overt. Once DMs have occurred, little or no attention will be paid to RR, and in addition, usually systemic treatment is started, which also influences subclinical RR. However, in this study, the absolute percentage of patients experiencing DM as the first event was only 4%, suggesting that the impact of the aforementioned problem is small. Furthermore, the CIF for RR as the first event was calculated with DM as the competing event taken into account.

Some strengths of this study were its population-based design and its large number of analyzed patients with complete data on first events. Use of an SLNB-negative cohort may be considered a weakness of the study. Ideally, a subset of SLNB-positive patients would have been used as well because these patients have a higher baseline risk for the development of RR. Then again, before 2010, the latter patients would have undergone routine ALND, and these patients later were considered candidates for RT of the axilla, in line with the results of the AMAROS trial. Another limitation of this study was that we had no detailed information on how RT treatment planning was done at the time that these patients were treated.

Many trials have provided evidence that breast cancer management often is too extensive, and a focus toward de-escalating treatment for a selection of patients has been proposed.^32^–^35^ In this study, we quantified the mitigating side effects of whole-breast RT, CT, and HT on RR incidence in a large cohort of SLNB N0 breast cancer patients. We demonstrated that the three described methods at least halved the risk. When we also take into account the historical finding of the NSABP-04 trial that residual metastatic lymph nodes will not automatically develop into a clinically detectable RR, even in the absence of the aforementioned therapies,^9^,^10^ the findings of the current study may help to explain the observed discrepancy between the false-negative rate of SLNB and regional recurrence in N0 patients. If we extrapolate the effect size of the nonsurgical treatments administered to SLNB N+ patients, the findings may even help the clinician better grasp the discrepancy between the rate of additional non-SLN (27%) and the observed RR rate (1.5%) when axillary clearance is omitted.

## Electronic Supplementary Material

Below is the link to the electronic supplementary material.

Fig. S1 Flowchart of the analyzed study population. SLNB, sentinel lymph node biopsy; RT, radiotherapy; BCT, breast-conserving therapy; ALND, axillary lymph node dissection (DOCX 29 kb)

## References

[1] Miltenburg DM, Miller C, Karamlou TB, Brunicardi FC (1999). Meta-analysis of sentinel lymph node biopsy in breast cancer. J Surg Res..

[2] Kim T, Giuliano AE, Lyman GH (2006). Lymphatic mapping and sentinel lymph node biopsy in early-stage breast carcinoma: a metaanalysis. Cancer..

[3] Pepels MJ, Vestjens JH, de Boer M (2011). Safety of avoiding routine use of axillary dissection in early-stage breast cancer: a systematic review. Breast Cancer Res Treat..

[4] van der Ploeg IM, Nieweg OE, van Rijk MC, Valdés Olmos RA, Kroon BB (2008). Axillary recurrence after a tumour-negative sentinel node biopsy in breast cancer patients: a systematic review and meta-analysis of the literature. Eur J Surg Oncol..

[5] van Wely BJ, Teerenstra S, Schinagl DA, Aufenacker TJ, de Wilt JH, Strobbe LJ (2010). Systematic review of the effect of external beam radiotherapy to the breast on axillary recurrence after negative sentinel lymph node biopsy. Br J Surg..

[6] Giuliano AE, Hunt KK, Ballman KV (2011). Axillary dissection vs no axillary dissection in women with invasive breast cancer and sentinel node metastasis: a randomized clinical trial. JAMA..

[7] Armando E, Giuliano AE, Ballman KV (2017). Effect of axillary dissection vs no axillary dissection on 10-year overall survival among women with invasive breast cancer and sentinel node metastasis: the ACOSOG Z0011 (Alliance) Randomized Clinical Trial. JAMA.

[8] Donker M, van Tienhoven G, Straver ME (2014). Radiotherapy or surgery of the axilla after a positive sentinel node in breast cancer (EORTC 10981-22023 AMAROS): a randomised, multicentre, open-label, phase 3 non-inferiority trial. Lancet Oncol..

[9] Roos MM, van Steenhoven JEC, Aalders KC (2019). Regional recurrence risk following a negative sentinel node procedure does not approximate the false-negative rate of the sentinel node procedure in breast cancer patients not receiving radiotherapy or systemic treatment. Ann Surg Oncol..

[10] Fisher B, Jeong JH, Anderson S, Bryant J, Fisher ER, Wolmark N (2002). Twenty-five-year follow-up of a randomized trial comparing radical mastectomy, total mastectomy, and total mastectomy followed by irradiation. N Engl J Med..

[11] van der Heiden-van der Loo M. Defining the Quality of Surgical Breast Cancer Care. Utrecht University 2013, Utrecht, the Netherlands.

[12] Moossdorff M, van Roozendaal LM, Strobbe LJ (2014). Maastricht Delphi consensus on event definitions for classification of recurrence in breast cancer research. J Natl Cancer Inst..

[13] van Wely BJ, Smidt ML, de Kievit IM, Wauters CA, Strobbe LJ (2008). False-negative sentinel lymph node biopsy. Br J Surg..

[14] van Wely BJ, van Wildenberg FJH, Gobardhan P (2012). Axillary recurrences after sentinel lymph node biopsy: a multicentre analysis and follow-up of sentinel lymph node-negative breast cancer patients. EJSO..

[15] Early Breast Cancer Trialists’ Collaborative Group, et al. (2011). Effect of radiotherapy after breast-conserving surgery on 10-year recurrence and 15-year breast cancer death: meta-analysis of individual patient data for 10,801 women in 17 randomised trials. Lancet..

[16] Rabinovitch R, Ballonoff A, Newman F, Finlayson C (2008). Evaluation of breast sentinel lymph node coverage by standard radiation therapy fields. Int J Radiat Oncol Biol Phys..

[17] Chung MA, DiPetrillo T, Hernandez S, Masko G, Wazer D, Cady B (2002). Treatment of the axilla by tangential breast radiotherapy in women with invasive breast cancer. Am J Surg..

[18] Reed DR, Lindsley SK, Mann GN (2005). Axillary lymph node dose with tangential breast irradiation. Int J Radiat Oncol Biol Phys..

[19] Van Roozendaal M, Schipper RJ, Smit L (2015). Three-dimensional breast radiotherapy and the elective radiation dose at the sentinel lymph node site in breast cancer. Ann Surg Oncol..

[20] De Santis MC, Bonfantini F, Dispinzieri M (2016). Axillary coverage by whole-breast irradiation in 1 to 2 positive sentinel lymph nodes in breast cancer patients. Tumori..

[21] Offersen BV, Boersma LJ, Kirkove C (2016). ESTRO consensus guideline on target volume delineation for elective radiation therapy of early-stage breast cancer, version 1.1. Radiother Oncol..

[22] Early Breast Cancer Trialists’ Collaborative Group (EBCTCG) (2005). Effects of chemotherapy and hormonal therapy for early breast cancer on recurrence and 15-year survival: an overview of the randomised trials. Lancet London England..

[23] Early Breast Cancer Trialists’ Collaborative Group (1992). Systemic treatment of early breast cancer by hormonal, cytotoxic, or immune therapy: 133 randomised trials involving 31,000 recurrences and 24,000 deaths among 75,000 women. Lancet London, England..

[24] Aalders KC, van Bommel ACM, van Dalen T (2016). Contemporary risk of local and regional recurrence and contralateral breast cancer in patients treated for primary breast cancer. Eur J Cancer..

[25] Howell A, Cuzick M, Baum M (2005). Results of the ATAC (arimidex, tamoxifen, alone or in combination) trial after completion of 5 years’ adjuvant treatment for breast cancer. Lancet..

[26] Mamounas EP, Bryant J, Lembersky B (2005). Paclitaxel after doxorubicin plus cyclophosphamide as adjuvant chemotherapy for node-positive breast cancer: results from NSABP-B28. J Clin Oncol..

[27] Baum M, Buzdar A, Cuzick J (2003). Anastrozole alone or in combination with tamoxifen versus tamoxifen alone for adjuvant treatment of postmenopausal women with early-stage breast cancer: results of the ATAC (arimidex, tamoxifen alone or in combination) trial efficacy and safety update analyses. Cancer..

[28] Martin M, Pienkowski T, Mackey J (2005). Adjuvant docetaxel for node-positive breast cancer. N Engl J Med..

[29] Mannino M, Yarnold JR (2009). Local relapse rates are falling after breast-conserving surgery and systemic therapy for early breast cancer: can radiotherapy ever be safely withheld?. Radiother Oncol..

[30] Lanning RM, Morrow M, Riaz N (2015). The effect of adjuvant trastuzumab on locoregional recurrence of human epidermal growth factor receptor 2-positive breast cancer treated with mastectomy. Ann Surg Oncol..

[31] Kiess AP, McArthur HL, Mahoney K (2012). Adjuvant trastuzumab reduces locoregional recurrence in woman who receive breast conservation therapy for lymph node-negative, human epidermal growth factor receptor 2-positive breast cancer. Cancer..

[32] Curigliano G, Burstein HJ, Winter PE (2017). De-escalating and escalating treatments for early-stage breast cancer. Ann Oncol..

[33] Blamey RW, Bates T, Chetty U (2013). Radiotherapy or tamoxifen after conserving surgery for breast cancers of excellent prognosis: British Association of Surgical Oncology (BASO) II trial. Eur J Cancer..

[34] Hughes KS, Schnaper LA, Bellon JR (2013). Lumpectomy plus tamoxifen with or without irradiation in women age 70 years or older with early breast cancer: long-term follow-up of CALGB 9343. J Clin Oncol..

[35] Kunkler IH, Williams LJ, Jack WJ, Cameron DA, Dixon JM (2015). Breast-conserving surgery with or without irradiation in women aged 65 years or older with early breast cancer (PRIME II): a randomised controlled trial. Lancet Oncol..

