# Supplementary material for: Quantifying the Mitigating Effects of Whole-Breast Radiotherapy and Systemic Treatments on Regional Recurrence Incidence Among Breast Cancer Patients
Source: Ann Surg Oncol. 2020 Mar 20;27(9):3402–11. doi: 10.1245/s10434-020-08356-2 (PMC7410865; doi:10.1245/s10434-020-08356-2)
Supplement: Supplementary file 1 — Flowchart of the analyzed study population. SLNB, sentinel lymph node biopsy; RT, radiotherapy; BCT, breast-conserving therapy; ALND, axillary lymph node dissection (DOCX 29 kb) [file 10434_2020_8356_MOESM1_ESM.docx]

**Supplementary figure 1.** Flowchart of the analysed study-population.

Dutch female patients diagnosed with primary invasive breast cancer (*stage I-II*) between 2005-2008

**n = 34,734**

Excluded from the analysis

SLNB not performed **n= 12,318**

SLNB performed **n= 22,416**

SLNB positive **n= 6,791**

Patients who underwent *neo-adjuvant therapy* (**n=0)**

Patients who underwent *mastectomy* and received *adjuvant RT* (**n=228)**

Patients who underwent *BCT* and *did not receive adjuvant RT* (**n=235)**

Patient who underwent *ALND* **(n=1,091)**

Patient staged as *pT4* (**n=94)**

Patients with *macro- or microscopic tumor residue* after final surgery (**n=** **465)**

SLNB negative **n= 15,625**

Dutch female *primary* invasive breast cancer (*stage I-II*) patients, treated by *BCT* followed by *adjuvant RT* or by *mastectomy without adjuvant RT* and who were staged as pN0 according to SLNB

**n = 13,512**

Abbreviations SLNB; sentinel lymph node biopsy, RT; radiotherapy, BCT; breast conserving therapy, ALND; axillary lymph node dissection
